# Development of interprofessional education programmes in nursing care and oral healthcare for dental and nursing students

**DOI:** 10.1186/s12909-024-05227-2

**Published:** 2024-04-08

**Authors:** Satoru Haresaku, Toru Naito, Hisae Aoki, Maki Miyoshi, Mayumi Monji, Yojiro Umezaki, Mami Miyazono, Rui Egashira, Akiko Chishaki

**Affiliations:** 1 Department of Nursing, Fukuoka Nursing College, 2-15-1 Tamura, Sawara-Ku, Fukuoka, 814-0193 Japan; 2 Section of Geriatric Dentistry, Department of General Dentistry, Fukuoka Dental College, 2-15-1 Tamura, Sawara-Ku, Fukuoka, 814-0193 Japan

**Keywords:** Interprofessional education, Oral healthcare, Nursing care, Dental students, Nursing students

## Abstract

**Background:**

Interprofessional education (IPE) is essential for healthcare students to collaborate effectively in multidisciplinary teams. This study aimed to identify the effect of IPE programmes in nursing care and oral healthcare on dental and nursing students’ perceptions of interprofessional collaboration.

**Methods:**

The study included 101 third-year dental and 98 fourth-year nursing students. The participants were divided into mixed-professional groups of four (2 dental and 2 nursing students). They participated in nursing care and oral healthcare training programmes that included student-on-student training and discussion groups. Questionnaires regarding perceptions of interprofessional collaboration were distributed to the participants before and after the programmes to compare the programmes before and after and between the dental and nursing students. The Wilcoxon signed-rank test and chi-square test were used to compare the data.

**Results:**

Data from 79 dental students (42 males and 37 females) and 89 nursing students (4 males and 85 females) who completed both questionnaires were used for the comparisons. Perceptions of the differences between the approaches of different health professionals to nursing care, the roles of other professionals, and the need for multiprofessional collaboration improved significantly among both dental and nursing students after the programmes. Although the perception of their ability to communicate with unfamiliar or new people improved significantly only among the nursing students, other perceptions of their ability to communicate did not improve for either group. More dental students than nursing students chose nursing trainings as good programmes to participate in with other professional students, while more nursing students than dental students chose oral care trainings as good programmes. Many students commented that they learned about nursing and oral healthcare skills as well as the importance of teamwork and communication with other professionals. Seven students commented that they were more motivated to become dentists and nurses.

**Conclusions:**

This study showed that IPE programmes for nursing care and oral healthcare might be effective at helping students understand other professionals and promoting multiprofessional collaboration. However, further studies are needed to develop IPE programmes to improve attitudes and abilities related to interprofessional communication skills.

## Background

Currently, approximately 15% of the population worldwide, or approximately 1 billion individuals, live with one or more disabling conditions. More than 46% of older people (over the age of 60) have disabilities, and more than 250 million older people experience moderate to severe disability [1]. Furthermore, these percentages and numbers are expected to increase as the percentage of older people in the global population increases from 12% in 2015 to 22% in 2050 [1, 2].

Nurses play an important role in providing nursing care for older people [3, 4]. In addition, they can perform oral assessments and make dental referrals through physicians [5, 6] and can perform collaborative oral healthcare with oral health professionals [7]. Oral health professionals can provide professional oral healthcare to prevent aspiration pneumonia and postoperative complications [8–10]. Therefore, both dental and nursing students need to learn about oral healthcare, nursing care, and interprofessional communication skills and to understand interprofessional healthcare practices [11].

Interprofessional education (IPE) is essential for healthcare students to collaborate effectively in multidisciplinary teams [12]. The World Health Organization (WHO) has consistently underscored the importance of IPE [13], and the World Dental Federation reported on IPE and collaborative practices in 2020 [14]. A study commissioned by the WHO reported that IPE is taking place in many countries, including Japan [15]. Among the many benefits of IPE, respondents note practice- and policy-related positive outcomes, such as improved access to care, health outcomes and quality of care, as well as workforce morale, practices and productivity. A survey of U.S. and Canadian dental schools indicated that the majority of schools that responded to the survey had established IPE programmes, most frequently with medical and nursing schools and dental hygiene programmes [16]. Several studies have shown that IPE between oral health professionals and nurses has a beneficial impact on students’ understanding of team medicine [17–22]. However, there are few studies and reports on IPE in Japan [11, 23]. Moreover, to our knowledge, no studies of IPE programmes have included a combination of nursing care training and oral healthcare training.

The purpose of this study was to identify the effect of IPE programmes in nursing care and oral healthcare on dental and nursing students’ perceptions of interprofessional collaboration.

## Methods

### Design and sample

**Fig. 1:**
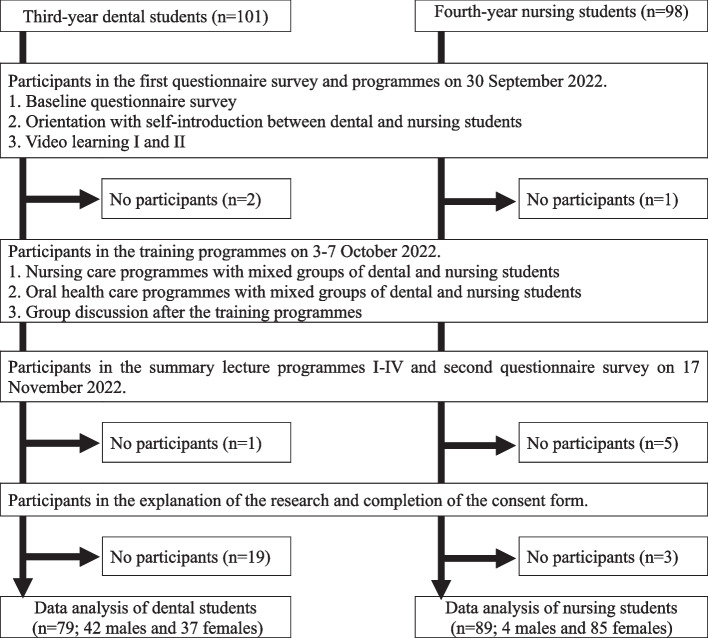
Flow chart illustrating the selection of study participants

This was a follow-up, cross-sectional study (Fig. 1). The participants were 101 third-year dental students in a 6-year dental school and 98 final-year nursing students in a fourth-year nursing school. All students were recruited for this study because the course was included in each school curriculum. The dental and nursing schools belong to the same school cooperative area in Fukuoka Prefecture, Japan. The nursing school enlists the cooperation of dental school in oral healthcare education.

Before the baseline study, the dental students had learned basic medical and dental sciences, but they had not learned subjects related to clinical dental work and had not participated in dental clinical training programmes. The nursing students had learned most nursing subjects, including the completion of 45-h oral healthcare programmes and clinical training programmes in hospitals and care facilities. The 45-h oral healthcare programmes were taught and instructed by multiple health professionals, such as dentists, dental hygienists, speech-language-hearing therapists, and nurses [24].

### Content of the IPE programmes

The nursing care and oral healthcare programmes were developed for dental students by dental faculty members [25] and were established as IPE programmes for dental and nursing students by the dental and nursing faculty members in this study. The programmes had four sections: orientation programmes that included two learning videos, nursing care training programmes, oral healthcare programmes, and four lectures (Table 1). In the video learning programmes, the students watched a video on nursing care skills and a video depicting a doctor struggling with various cases in home healthcare. In the nursing training programmes, the students performed student-on-student training in basic nursing skills. In addition, they wore old-age simulation suits (Sakamoto Model Ltd., Kyoto, Japan) to experience the joint limitations, muscle weakness, and visual field narrowing that elderly people experience. In the oral healthcare programmes, they participated in training on denture removal and fitting using an oral simulator with full dentures in the mandible and partial dentures in the maxilla (Sakamoto Model Ltd., Kyoto, Japan). In addition, they underwent training in student-on-student oral healthcare. In the lectures, three physicians and a dentist used slides to provide information on practices and healthcare for elderly people. After the training programmes, self-introduction between dental and nursing students in the orientation programme (P1) and a group discussion programme (P11) was added to promote interprofessional communication.

**Table 1:** Content of the IPE programmes

Programme	Time (Minutes)	Content
P1. Orientation	80	• Providing an overview of the programmes to the students and self-introduction of dental and nursing students
P2. Video I; have learned I	40	• Watching a video on nursing care skills
P3. Video I; have learned II	120	• Watching a video about the struggles of doctors dealing with various cases in home healthcare and thinking about aspiration pneumonia and euthanasia in elderly individuals
P4. Nursing care training I	30	• Watching a video of a physician performing cognitive function screening tests with an elderly person with dementia, trying the tests themselves, and evaluating the results
P5. Nursing care training II	30	• Performing student-on-student training in assisting patients in walking with a cane
P6. Nursing care training III	45	• Performing student-on-student training in transferring patients from wheelchair to bed
P7. Nursing care training IV	55	• Performing student-on-student training on changing the patient’s position in bed
P8. Experiencing the old age simulation suits	45	• Wearing an old age simulation suit and experiencing joint limitations, muscle weakness, and visual field narrowing
P9. Oral healthcare training I	15	• Performing training on denture removal and fitting with an oral simulator
P10. Oral healthcare training II	45	• Performing student-on-student training of oral healthcare
P11. Group discussion after the training programmes	30	• Discussing what has been learned through the training programmes
P12. Summary lecture I	80	• Lecture on the pathology of cardiovascular disease and preventive measures for patients with the disease by physician A
P13. Summary lecture II	80	• Lecture on the rehabilitation of patients with paralysis by physician B
P14. Summary lecture III	80	• Lecture on the main legal and financial accounts relating to the period between a person’s birth and death by a dentist
P15. Summary Lecture IV	80	• Lecture on the actual practice of home healthcare by physician C

The dental and nursing students were divided into four groups and were further subdivided into mixed-professional subgroups of four (2 dental and 2 nursing students). The nursing care and oral healthcare training programmes were conducted on separate days for each group, and student-on-student training was performed within the subgroup.

The orientation and lecture programmes were conducted in a hall with a 600-seat capacity on the same grounds. The nursing care training programmes were conducted in a training room in the nursing school, and the oral healthcare training programmes were conducted in the affiliated hospital.

### Structured questionnaires

The first paper-based questionnaire was based on a previously developed questionnaire used to assess the effectiveness of IPE programmes on improving students’ perceptions of interprofessional collaboration [26].

The questionnaire consisted of 2 parts (Table 2): self-perceptions of one’s skills in communicating with other health professionals (Q1-6) and understanding of the role of other health professionals and the need for interprofessional collaboration (Q7-12). A four-point Likert response scale was used for each item, with choices including “agree”, “somewhat agree”, “somewhat disagree”, and “disagree”. These response choices for the question items were scored as “4”, “3”, “2”, and “1” for all items except Q1, Q3, and Q7. The latter questions were negative and reverse scored, and the response options for those items were “1”, “2”, “3”, and “4”; therefore, a score of 4 indicated the highest level of positive perception.

**Table 2:** Questionnaire items on perceptions of interprofessional collaboration practice

Self-perceptions regarding communication ability with other health professionals
Q1. I am not good at communicating with unfamiliar or new people^a^.
Q2. I can actively communicate with others on my own initiative.
Q3. I am not good at articulating my opinions to others^a^.
Q4. I can listen to and examine opinions that differ from my own.
Q5. I can support members of the same group.
Q6. I can review, self-evaluate, and manage my own behaviour during the group activities.
Understanding other health professionals and collaborative healthcare
Q7. I do not know the differences between the approaches of different health professionals to nursing care^a^.
Q8. I can consider a holistic approach to elderly people with regard to psychological and social factors.
Q9. I have respect for other health professionals.
Q10. I believe that a healthcare team is necessary for the health professional I aspire to become.
Q11. I understand the role of other health professionals in the healthcare team.
Q12. I understand the role of the health professional I aspire to become in the healthcare team.

^a^Negative and reverse designed items

The second web-based questionnaire survey was conducted via Google Forms (Google LLC, California, USA). Three items related to the students’ opinions about the programmes were added to the items in the first questionnaire: a) “Which programmes were good to participate in?”, b) “Which programmes were good to participate in together with other professional students?”, and c) “What were the positive and negative aspects of participating in the IPE programme with other professional students?”.

The students chose only 3 out of 15 programmes for questions a) and b), and they provided their comments for question c). Two researchers, who were nurses (M.M1 and M.M2), had worked more than 10 years as nursing faculty members and were experts in qualitative data analysis, read all the descriptive comments in question c) and coded the sentences based on their similarities. The researchers subcategorized the sentences into 30 and 28 groups according to the dental and nursing students’ comments, respectively. The sentences were further categorized into 14 and 15 groups, respectively. Finally, a researcher and dentist who had worked for 15 years as a dental faculty member and 6 years as a nursing faculty member checked the analysis to ensure validation.

McDonald’s omega was used to assess the reliability of the questionnaire items with regard to perceptions of interprofessional collaboration practices in the first and second questionnaires. McDonald’s omega coefficients for students’ perceptions of interprofessional collaboration before and after the programmes were 0.740 and 0.773, respectively.

### Data procedure

The first questionnaire survey was conducted immediately before the orientation programme on 30 September 2022, and the second was conducted immediately after the summary lecture programmes on 17 November 2022 (Fig. 1). Data from students who did not participate in the explanation of the study or did not complete the consent form were excluded from the study.

All procedures involving human participants were approved by the Ethics Committee of Fukuoka Gakuen, Fukuoka, Japan (approval no. 612) and were in accordance with the Ethical Guidelines for Clinical Research (the Ministry of Health, Labour and Welfare, Tokyo, Japan, no. 415 of 2008) and the 1964 Declaration of Helsinki and its later amendments or comparable ethical standards.

### Statistical analyses

The Kolmogorov‒Smirnov test was used to confirm the normality of the data. Since the data were confirmed to not be normally distributed, a Wilcoxon signed-rank test was used to compare students’ perceptions before and after the programmes, and a Mann‒Whitney U test was used to compare dental students’ and nursing students’ perceptions. A chi-squared test or Fisher’s exact test was used to compare the differences between dental and nursing students in terms of the percentage of responses regarding whether the students felt that the programmes were good.

Data were analysed at the 5% significance level. Statistical analyses were performed using the IBM SPSS Statistics software program (version 28.0; IBM Corporation, Armonk, NY, USA).

## Results

A total of 79 (78.2%) dental students and 89 (90.8%) nursing students participated in the surveys and completed the consent form (Fig. 1). Most of the dental students were male (68.1%), and most of the nursing students were female (95.5%). The mean age at baseline was 23.1 ± 3.8 years for dental students and 21.8 ± 0.9 years for nursing students.

Table 3 shows a comparison of the dental students’ and nursing students’ perceptions of interprofessional collaboration before and after the programmes. The range of the mean perception level at baseline was 2.27–3.73 for the dental students and 2.24–3.91 for the nursing students. The total perception levels were significantly greater for the nursing students than for the dental students both before and after the programmes (*P* < 0.05). The perception levels of the dental students for Q7, Q8, Q11, and Q12 and those of the nursing students for Q1, Q6-8, Q11, and Q12 were significantly greater after the programmes than before (*P* < 0.05).

**Table 3:** Comparison of perception levels (1–4)^a^ of interprofessional collaboration before and after the programmes

	Dental students (*n* = 79)			Nursing students (*n* = 89)			Dental vs. nursing students	
	Before	After	*P* value*	Before	After	*P* value*	Before	After
	Mean (SD)	Mean (SD)		Mean (SD)	Mean (SD)		*P* value**	*P* value**
Q1. I am not good at communicating with unfamiliar or new people^a^.	2.46 (0.94)	2.61 (0.95)	0.073	2.45 (0.84)	2.66 (0.89)	0.004	0.930	0.665
Q2. I can actively communicate with others on my own initiative.	2.62 (0.85)	2.73 (0.86)	0.141	2.66 (0.74)	2.69 (0.78)	0.525	0.803	0.685
Q3. I am not good at articulating my opinions to others^a^.	2.38 (0.92)	2.42 (0.79)	0.745	2.24 (0.80)	2.35 (0.85)	0.178	0.264	0.597
Q4. I can listen to and examine opinions that differ from my own.	3.25 (0.69)	3.29 (0.66)	0.653	3.54 (0.54)	3.58 (0.56)	0.468	0.006	0.003
Q5. I can support other members of the same group.	3.37 (0.60)	3.32 (0.63)	0.450	3.61 (0.54)	3.58 (0.58)	0.683	0.007	0.003
Q6. I can review, self-evaluate, and manage my own behaviour during group activities.	3.10 (0.78)	3.18 (0.59)	0.300	3.35 (0.62)	3.51 (0.52)	0.013	0.041	0.000
Q7. I do not know the differences between the approaches of different health professionals to nursing care^a^.	2.27 (0.80)	3.25 (0.71)	0.000	2.73 (0.65)	3.31 (0.72)	0.000	0.000	0.488
Q8. I can consider a holistic approach to elderly people, including psychological and social factors.	3.00 (0.68)	3.19 (0.64)	0.035	3.22 (0.52)	3.47 (0.50)	0.000	0.031	0.005
Q9. I have respect for other health professionals.	3.73 (0.57)	3.78 (0.47)	0.601	3.87 (0.38)	3.92 (0.27)	0.132	0.104	0.031
Q10. I believe that a healthcare team is necessary for the health professional I aspire to become.	3.72 (0.55)	3.84 (0.37)	0.074	3.91 (0.32)	3.96 (0.21)	0.206	0.004	0.011
Q11. I understand the role of other health professionals in the healthcare team.	3.05 (0.77)	3.46 (0.57)	0.000	3.30 (0.53)	3.58 (0.50)	0.000	0.043	0.172
Q12. I understand the role of health professional I aspire to become in the healthcare team.	3.13 (0.70)	3.52 (0.50)	0.000	3.51 (0.52)	3.73 (0.45)	0.000	0.000	0.005
Total (level 12–48)	36.08 (5.06)	38.58 (4.38)	0.000	38.38 (3.41)	40.35 (3.96)	0.000	0.001	0.043

^*^Wilcoxon Signed-rank Test

^**^Mann‒Whitney U Test

^a^A score of 4 indicated the highest level of positive perceptions

Table 4 shows the distribution of programmes that the students indicated were good programmes to participate in or were good to participate in with other professional students (choosing the top 3). Among the types of programmes, the largest percentage of students indicated that it was good to participate in student-on-student oral healthcare training (P10, 52.4%), with percentages of 40–50% for P3, P8, and P15. The percentages of students who selected P1, P5, P6, and P7 were significantly greater among dental students than nursing students (*P* < 0.05), although the percentage of students indicating P9 was significantly lower among dental students than nursing students (*P* < 0.001). The type of programme with the largest percentage of student support for participation with other professional students was training in transferring patients from wheelchairs to beds (P7, 59.4%), which showed percentages for P5 and P7 of 40–50%. The percentages for P5, P6, and P7 were significantly higher for dental students than nursing students (*P* < 0.001), although the percentages for P9, P10, and P11 were significantly higher for nursing students than for dental students (*P* < 0.05).

**Table 4:** Distribution (%) of programmes selected as good programmes to participate in or to participate in with other professional students

Programme	Good programmes to participate in (Choose the top 3)				Good programmes to participate in with other professional students (Choose the top 3)			
	Total (*n* = 168)	Dental students (*n* = 79)	Nursing students (*n* = 89)	*P* value^*^	Total (*n* = 168)	Dental students (*n* = 79)	Nursing students (*n* = 89)	*P* value^*^
	n (%)	n (%)	n (%)		n (%)	n (%)	n (%)	
P1. Orientation and self-introduction	8 (4.8)	7 (8.9)	1 (1.1)	0.027	27 (16.1)	15 (19.0)	12 (13.5)	0.332
P2. Video I; have learned I	3 (1.8)	3 (3.8)	0 (0.0)	0.102	3 (1.8)	3 (3.8)	0 (0.0)	0.102
P3. Video I; have learned II	75 (44.6)	36 (45.6)	39 (43.8)	0.820	6 (3.6)	3 (3.8)	3 (3.4)	1.000
P4. Performing cognitive function tests	31 (18.5)	11 (13.9)	20 (22.5)	0.154	13 (7.7)	5 (6.3)	8 (9.0)	0.520
P5. Training in assisting patients to walk with a cane	30 (17.9)	21 (26.6)	9 (10.1)	0.005	68 (40.5)	47 (59.5)	21 (23.6)	0.000
P6. Training in transferring patients from a wheelchair to bed	36 (21.4)	23 (29.1)	13 (14.6)	0.022	100 (59.5)	59 (74.7)	41 (46.1)	0.000
P7. Training in changing the patient’s position in bed	26 (15.5)	17 (21.5)	9 (10.1)	0.041	80 (47.6)	53 (67.1)	27 (30.3)	0.000
P8. Experience wearing age simulation suits	75 (44.6)	32 (40.5)	43 (48.3)	0.310	52 (31.0)	24 (30.4)	28 (31.5)	0.880
P9. Training in the fitting and removal of dentures using a denture model	39 (23.2)	6 (7.6)	33 (37.1)	0.000	43 (25.6)	3 (3.8)	40 (44.9)	0.000
P10. Training in oral healthcare practice	88 (52.4)	38 (48.1)	50 (56.2)	0.295	65 (38.7)	8 (10.1)	57 (64.0)	0.000
P11. Group discussion after the training programmes	7 (4.2)	3 (3.8)	4 (4.5)	1.000	20 (11.9)	5 (6.3)	15 (16.9)	0.036
P12. Summary lecture I	12 (7.1)	6 (7.6)	6 (6.7)	0.830	2 (1.2)	1 (1.3)	1 (1.1)	1.000
P13. Summary lecture II	20 (11.9)	9 (11.4)	11 (12.4)	0.847	6 (3.6)	3 (3.8)	3 (3.4)	1.000
P14. Summary lecture III	7 (4.2)	4 (5.1)	3 (3.4)	0.708	2 (1.2)	0 (0.0)	2 (2.2)	0.499
P15. Summary lecture IV	74 (44.0)	33 (41.8)	41 (46.1)	0.576	11 (6.5)	5 (6.3)	6 (6.7)	0.914

^*^Chi-square test or Fisher’s exact test

Table 5 shows the comments from dental and nursing students on the IPE programmes. The dental students commented that they “have learned many things from new experiences” (*n* = 17), “have learned many things from nursing students” (*n* = 14), “have learned nursing skills from nursing students” (*n* = 11), “have learned the needs and importance of teamwork” (*n* = 10), and “could understand other professionals” (*n* = 10). In addition, all their comments were positive. The nursing students commented that they “gained confidence in performing oral healthcare” (*n* = 25) and “were able to review nursing care skills” (*n* = 23). In addition, they commented that they “have learned many things through mutual teaching interprofessionally” (*n* = 22) and learned “from new perspectives” (*n* = 20) and “from new experiences” (*n* = 19). However, some nursing students had negative comments, such as “the programmes were not appropriate for fourth-year nursing students but might be good for the lower-year nursing students” (*n* = 5), “some students were not taking the trainings seriously” (*n* = 3), and “there was nothing to be taught by dental students” (*n* = 3).

**Table 5:** Comments from dental and nursing students on the IPE programmes

Dental students’ comments	n (%)	Nursing students’ comments	n (%)
• I have learned many things from new experiences.	17 (21.5)	• I gained confidence in performing oral healthcare.	25 (28.1)
• I have learned many things from nursing students.	14 (17.7)	• I was able to review my nursing skills.	23 (25.8)
• I have learned nursing skills from nursing students.	11 (13.9)	• I have learned many interprofessional skills through mutual teaching.	22 (24.7)
• I have learned the necessity and importance of team medicine.	10 (12.7)	• I have learned many things from new perspectives.	20 (22.5)
• I can understand other professionals.	10 (12.7)	• I have learned many things from new experiences.	19 (21.3)
• I have learned interprofessional skills through mutual teaching.	9 (11.4)	• I have learned oral health from dental students and academic staff.	17 (19.1)
• I have learned many things from new perspectives.	6 (7.6)	• I have learned the needs and importance of team medicine.	14 (15.7)
• I realized communication difficulties with other professional students.	5 (6.3)	• I can understand other professionals.	5 (5.6)
• I am aware of the importance of communication with other professional students.	4 (5.1)	• I feel that the programmes are not appropriate for fourth-year nursing students but are good for lower-year nursing students.	5 (5.6)
• I hope to have further exchanges with nursing students.	4 (5.1)	• I am more motivated to become a nurse.	4 (4.5)
• I am more motivated to become a dentist.	3 (3.8)	• I am aware of the importance of communication with other professional students.	3 (3.4)
• I hope to learn more about nursing.	3 (3.8)	• I am interested in a lecture regarding the practice of home healthcare.	3 (3.4)
• I hope to learn about nursing with real patients and facilities.	3 (3.8)	• I am aware that some students do not take the trainings seriously.	3 (3.4)
• Other^a^	21 (26.6)	• I perceive that there is nothing to be taught by dental students.	3 (3.4)
		• Other^a^	18 (20.2)

^a^Fewer than 2 comments

## Discussion

This follow-up and cross-sectional study was the first to report on IPE programmes that included student-on-student nursing care and oral healthcare trainings and their effectiveness in improving students’ perceptions of multiprofessional collaboration. The results showed that at baseline, the nursing students’ perception levels were higher than those of dental students. The fourth-year nursing students had already earned credits in most nursing subjects and had experienced clinical nursing care in hospitals and care facilities. In addition, the nursing students in the nursing school had completed 45-h oral health and healthcare courses that could improve their level of perception of the importance of collaboration with healthcare workers in oral healthcare practice [24]. Conversely, the dental students had earned credits only basic medical and dental subjects and had not experienced clinical dental practice. Therefore, the differences in learning and experiences between dental and nursing students might affect the differences in their perception levels at baseline.

After the IPE programmes, both dental and nursing students’ levels of perception of their understanding of other health professionals, collaborative healthcare, and the role of professionals that they aspired to achieve improved. Previous studies on IPE programmes have shown significant improvements in knowledge and perceptions of oral health and oral healthcare among nursing students [17–22]. A previous study reported that nursing students had low perceptions of oral health before the implementation of the IPE programme [22]. The present study might be the first to show that the IPE programme was effective at improving the perceptions of nursing students who already had a high level of education in nursing and oral healthcare [27] and improving dental students’ understanding of other professionals through nursing care training programmes.

The level of perceived interprofessional communication skills did not improve significantly among the dental students, although the levels improved slightly for some of the nursing students. The discussion time in the programmes was only 30 min, which might have been insufficient to improve the students’ perceptions. A previous comparison of IPE programmes between nursing and dental hygiene students reported that both student groups enjoyed working with each other, sharing skill sets and experiencing each other’s professional language [17]. In this study, approximately 70% of the dental students were male and almost all the nursing students were female. Therefore, the difference in the sex ratio in this study might be a barrier to interprofessional communication, although further studies are needed to prove this finding. Moreover, because the nursing programmes were developed by academic dental staff, dental students might not have a good understanding of this aspect of nursing, which might contribute to their lack of improved confidence in their interprofessional communication skills. A previous study reported that an interprofessional problem-based learning programme for medical and nursing students was effective at improving students’ ability and attitudes with regard to interprofessional communication and collaboration [28]. Therefore, IPE programmes with problem-based learning programmes should be developed in collaboration with dental and nursing staff to improve their perceptions.

Programmes that involved student-on-student oral health care training, the use of age simulation suits, the use of a film depicting the struggles of home healthcare doctors, and home healthcare doctors’ lectures were popular among both dental and nursing students. A programme using age simulation suits was effective at reducing negative attitudes towards elderly people in caregiving settings and enhancing empathy and role-taking in relation to this population [29]. Therefore, such programmes might increase students’ interest in home healthcare practice and geriatric medicine. For dental students, the programmes that were selected as good programmes to participate in with other professional students were nursing skill training programmes, but for nursing students, they were oral healthcare programmes. The results showed that students’ satisfaction with other professional skills programmes might improve if they teach these programmes to each other in IPE. These programmes might also improve students’ understanding of other health professionals.

The comments from the dental and nursing students on the IPE programmes revealed that the students had learned many things, such as the difficulties and importance of communicating with students in other professions, oral healthcare skills, and nursing care skills cultivated through mutual teaching, as new experiences from new perspectives. This new understanding might contribute to improving students’ perceptions of interprofessional collaboration practices. However, there were some negative comments among the nursing students in the programmes. As mentioned above, there were significant differences between the dental and nursing students with regard to their learning status as professional subjects. Many fourth-year dental students were not able to participate in this study because they needed to complete rounds in each dental clinical department or dental hospital in small groups and lacked the time to participate in the programmes throughout the school year. Differences in their learning and experience of their professional subjects might have affected the nursing students’ negative comments and improvements to their perception of interprofessional collaboration practices. Therefore, the coordinators of IPE programmes should consider students’ learning status when they choose the participants in programmes.

There are several limitations associated with this study. First, one Japanese dental school and one Japanese nursing school were investigated, and the sample of students was selected without a power calculation. There were 29 dental schools and 267 nursing schools in Japan at the time of the study [30]. The results of this study are therefore not generalizable. Second, perceptions of collaboration with other healthcare professionals might have been greater for these nursing students than for other nursing students because these students had completed 45-h oral healthcare programmes before the present study [24]. Third, 78.2% of the dental students and 90.8% of the nursing students participated in the study. This difference might have affected the results of this study because people interested in the topic were more likely to respond than people who were not interested [31]. Fourth, there were differences in the knowledge and experiences of the dental and nursing students. IPE for dental and nursing students with the same levels of knowledge and experience might be more effective at improving their perceptions of interprofessional collaboration practices. Fifth, the accuracy and relevance of the videos were not validated. However, 44.6% of the students chose video learning II as a good programme for participation. This programme might have affected their understanding of elderly care nursing. Finally, the same Likert scale was used in the previously developed questionnaire to assess the effectiveness of IPE programmes on the improvement of students’ perceptions of interprofessional collaboration [24]. However, the small range of the scale (Levels 1–4) might lead to a small estimate of the impact of the programmes on improving perceptions of interprofessional collaboration practices.

Despite several limitations of this study, the advantage of this study compared to previous IPE studies involving dental and nursing students is that teaching their professional skills to each other may provide students with a better understanding of interprofessional collaboration practices than learning them separately.

## Conclusions

This study showed that IPE programmes in nursing care and oral healthcare might be effective at improving the understanding of other professionals and multiprofessional collaboration among dental and nursing students but not at improving interprofessional communication skills among dental students. However, further studies are needed to develop IPE programmes to improve attitudes and abilities related to interprofessional communication skills.

## Data Availability

The datasets generated and/or analysed during the current study are not publicly available as ethics approval was granted on the basis that only the researchers involved in the study could access the identified data. However, the data are available from the corresponding author upon reasonable request.

## Abbreviation

IPE: Interprofessional education
